# A National Quality Initiative to Improve Palliative Care Outcomes: Identifying Enabling Factors that Drive Quality Improvement

**DOI:** 10.1089/pmr.2024.0092

**Published:** 2025-05-09

**Authors:** Sabina Clapham, Katherine Clark, Kylie Draper, Fiorina Mastroianni, Jesse Rand, Lisa Redwood, David Currow

**Affiliations:** ^1^ Palliative Care Outcomes Collaboration, University of Wollongong, Wollongong, Australia.; ^2^ Northern Sydney Local Health District, Supportive and Palliative Care Network, St Leonards, Australia.; ^3^ Northern Clinical School, The University of Sydney, Camperdown, Australia.; ^4^ Improving Palliative, Aged and Chronic Care through Clinical Research and Translation, Broadway, University of Technology Sydney, Sydney, Australia.

**Keywords:** education, improvement framework, national, palliative care, quality improvement

## Abstract

**Background:**

The Palliative Care Outcomes Collaboration (PCOC), established in 2005 and funded by the Australian Government, is a national quality improvement initiative that integrates patient outcome measures into routine clinical practice. While PCOC supports services to improve patient care, implementation across diverse clinical settings presents challenges, with variation observed between similarly resourced services. Engaging services in continuous quality improvement proves difficult as the program grows.

**Objectives:**

To identify factors associated with high-performing palliative care services and develop and evaluate an implementation framework and education program that supports continuous quality improvement.

**Methods:**

Patient outcome data and case studies from established PCOC-participating services were analyzed to identify high-performing services and the factors enabling successful integration of outcome measures. Based on the findings, an implementation framework was developed. Improvement trends were assessed in 20 services participating pre-intervention (2016–2018) and 11 services participating post-intervention (2022–2024).

**Results:**

Five key strategies and 25 enabling factors for successful integration were identified including, leadership and governance; education to improve data literacy; infrastructure for the meaningful management of data; and uptake of PCOC in quality systems. The post-intervention services started with higher benchmark performance and showed improvements within 6 months of implementation. Addressing patient’s psychological/spiritual needs continues to be challenging.

**Conclusion:**

Quality improvement involving outcome measurement and benchmarking in palliative care requires education and structured implementation with ongoing feedback. The PCOC initiative demonstrates that improving patient outcomes involves more than collecting and analyzing outcome measures and benchmarking—it requires integrated assessment models, education, and resources to support information-driven quality improvement.

## Introduction 

Measurement of patient outcomes is fundamental to improving the quality of clinical care. Particularly for people with life-limiting illnesses, yet implementation of routine outcome measurement presents many challenges.^1,2^ While it is feasible to measure outcomes on a national scale, its effectiveness depends on integration within the health system and the clinical, professional, and organizational support it receives.^3^ The Palliative Care Outcomes Collaboration (PCOC) in Australia exemplifies a national outcome and benchmarking program. PCOC aims to embed point-of-care patient-(and, at times, proxy-) reported outcomes across all palliative care settings. This initiative seeks to enhance care planning and drive outcome improvements through feedback and peer-to-peer benchmarking.^4^

PCOC commenced as a national Australian initiative in 2005. The first inception and feasibility study was published in 2008,^5^ and the establishment of the PCOC model, selection of tools, and development of the outcome measures and benchmarks was published in 2010.^4^ An estimated 75% of all public or privately funded Australian specialist palliative care services had voluntarily joined the initiative by that time. Training on the assessment tools and change in service culture to embed the measures were identified as key to implementation fidelity.^4^ An update, explaining the role of the external PCOC facilitator was published in 2011.^6^ The growth of the program and an analysis of improvements in patient outcomes was published in 2014,^7^ and a case study report summarizing the PCOC intentions was published in 2018.^8^ By 2021, PCOC represented 25% of all patients who might benefit from palliative care.^9^ As of July 2024, 197 services are voluntarily participating in the PCOC quality initiative, reporting on the outcomes of 70,000 patients each year.^10^

The implementation of outcome measures and sustaining a culture of continuous improvement remains challenging and variations in outcomes between similarly resourced palliative care services persist. For example, in 2023 a 5-fold variation in pain outcomes was reported nationally.^11^ While well-established implementation and improvement interventions such as education,^12^ and audit and feedback,^13^ have been integral to the PCOC initiative, a framework for preparing services to implement PCOC was lacking. This study aims to develop an implementation framework based upon case studies of high-performing services and to integrate this new framework into the education program to support new services joining the PCOC quality initiative. A preliminary review of the new framework was evaluated by comparing the starting benchmark score of new PCOC services pre and post-implementation, reviewing their benchmarks over time, and comparing the difference in scores after 2 years.

### Setting and participants

Palliative care in Australia is available in all states and territories in most health care settings. It is predominantly funded through a universal insurance scheme, Medicare, and provided in hospital, home, outpatient, residential aged care, correctional and disability settings. Australia has a well-established national safety, quality standards, and health service accreditation system. Standards and accreditation processes sit alongside National Palliative Care Standards,^14^ and a National Palliative Care Strategy.^15^ PCOC has been integrated into this system since 2005. PCOC is voluntary and available to all services providing palliative care. Participants include patients in receipt of palliative care. As of June 2024, most patients were diagnosed with a malignant principal-life limiting illness (61.7%), with a median age of 75 years, and identifying as male (52.6%). In addition, 64.0% of participants were Australian born, and 90.0% prefer English as their primary language. Over the study period, there was a notable increase in the proportion of patients with nonmalignant principal life-limiting illness, rising from 26.1% in 2018 to 36.4% in 2024.

## Methods

The primary aim of PCOC is to improve the quality of clinical care for patients with life-limiting illnesses through systematically collected point-of-care data which is analyzed centrally and fed back as value added information that can be benchmarked. Participating palliative care services submit their routinely collected patient data to PCOC and these data are used to evaluate palliative care outcomes. The routinely collected PCOC data has three levels: patient, episode, and phase. The patient level data mainly includes demographic characteristics. Episode level data contains information related to the period a patient spends within a care setting. Phase level data involves clinical assessment of the patient and family using five tools.^16^ The five PCOC tools include the Symptom Assessment Scale (SAS), Palliative Care Problem Severity Score (PCPSS), Australia-modified Karnofsky Performance Scale (AKPS), and the Resource Utilisation Groups—Activities of Daily Living (RUG-ADL), and Palliative Care Phase.^17–21^ When a symptom or problem is assessed, it is classed as absent to mild or moderate to severe. The PCOC benchmarks are assessed on the percentage of palliative care phases in absent to mild that ended in absent to mild for each symptom and the percentage of patients that commenced the palliative care phases in moderate to severe that ended in the phase absent to mild for each symptom (SAS and PCPSS). In addition to patient-level data, participating services provide service-level data including the location, size, care settings and delivery model, multidisciplinary team configuration, data collection systems and processes, and other implementation related factors.

### Study design

This study employed a mixed-methods design that combined quantitative analysis of patient outcome data with qualitative and quantitative case studies to identify factors contributing to high-performing palliative care services. These data were used to develop and refine an implementation framework to support the central principle of routine assessment and response underpinning outcome measurement. The intervention of interest is a complex education program to systematically implement PCOC and drive rapid, measurable quality improvement in participating services.

This study was conducted in three stages and reported following the Standards for Quality Improvement Reporting Excellence (SQUIRE 2.0) guideline.^22^ Stage one involved a retrospective quantitative analysis of benchmark results in 2019 to identify high-performing participating services. Stage two used case studies combining quantitative and qualitative service-level data from PCOC with insights from PCOC Improvement Facilitators and clinical champions (e.g., managers and clinical leads). In stage three, a refined implementation framework introduced from April 2021 was evaluated through quantitative analysis of benchmark results, comparing services that commenced PCOC between 2016 and 2018 with services starting in 2022.

### Theoretical underpinnings of the PCOC quality initiative

The PCOC initiative draws upon complexity science which acknowledges the need for adaptive change at multiple, interacting levels.^23^ Theory of Change,^24^ guides and supports PCOC core interventions at national and local levels. It provides PCOC with a pragmatic framework to identify and articulate interventions and measure their impact. The Precede-Proceed,^25,26^ model operationalizes how PCOC’s Theory of Change translates into action, reflecting the dynamic and complex nature of implementing outcome measurement in palliative care, and recognizing predisposing, enabling and reinforcing factors at the systems, service, and clinician level that are sequential and required to effect, sustain and embed change.

### Stage one: identifying high-performing services

Patient outcome data from palliative care services participating in PCOC for more than 4 years were analyzed. Services with patient-reported benchmarked measures in the top 25% of all participating services were identified as high-performing.^27^ Multiple case study methodology as described by Yin was employed and embedded,^28^ due to the suitability for ‘real-world’ situations. The multiple design refers to the number of case studies and embedded refers to the analysis of quantitative and qualitative data within each case. The unit of analysis is a palliative care service, and the phenomenon of interest is performance against benchmarks in the year 2019. A data extraction form was developed including service characteristics (setting, model, size, and location); factors predisposing the service to achieve benchmarks (e.g., leadership engagement, dedicated education and quality staff, and data infrastructure); factors enabling the service (e.g., education, assessment workflows that include the multidisciplinary team); and factors reinforcing the service (e.g., improved patient outcome/results, normalization of quality improvement).^26^ This information on palliative care services is routinely collected as part of supporting participation. PCOC facilitators responsible for supporting each service provided additional insights. Managers and clinical leaders from high-performing services were consulted to confirm the findings or to provide further details. Responses were analyzed descriptively.

### Stage two: developing the implementation framework and integrating it into PCOC’s education program

The identified enabling factors of high-performing services were grouped and organized into a framework by SC and FM. This framework was then integrated into PCOC’s routine support and education to provide structured readiness and preparation for services in the 6 months prior to participation in benchmarking reports by SC and PCOC improvement facilitators. The education program and implementation approach were made available for all participating services and systematically introduced and applied to newly participating services.

### Stage three: preliminary analysis of the implementation framework

Newly participating services were selected to evaluate the intervention through quantitative analysis of their benchmark results. This population was selected to ensure the exclusive use of the new implementation framework and education program. Recent increases in funding for palliative care clinical services across most Australian States and Territories, have allowed for the development of new and existing services to expand their delivery model and to commence using PCOC. Implementing the new framework in services that have been part of the initiative for several years would introduce complexity and was therefore out of scope for this study.

A pre- and post-intervention cohort was defined to allow a comparison group. The pre-intervention cohort included services that received their first PCOC report between 2016 and 2018 and had received four consecutive reports. The post-intervention cohort included services that had received their first PCOC report in 2022 and had received four consecutive reports. To assess the preliminary results of the implementation framework the percent of phases that met the PCOC benchmarks were compared between 6 months and 24 months of participation in PCOC in each group and between the pre- and post-intervention groups.

The pre-intervention period was selected as 2016–2018, so the fourth PCOC report was produced prior to the effects of the Coronavirus (COVID-19). The years 2019 and 2021 were also excluded due to the impact of COVID-19 on palliative care services which may alter the finding of this study.^29,30^ The pre-intervention period consists of the years where there was a significant advancement in implementation science applied across all participating services by the PCOC initiative. However, interventions were not specifically focused on service readiness and preparation for participation and not integrated into the education program. The analysis covered a 2-year period—four reporting cycles, for each service, to capture only the first 24 months of participation in PCOC for both groups. This included services commencing PCOC between January and December 2022. Particular attention to trends in outcome improvement and rate of improvement following the introduction of the framework and education program were examined. Data analysis was conducted using R studio.

Ethics the PCOC: A voluntary program to improve palliative care patient and carer outcomes in Australia has been reviewed and approved by the Greater Western New South Wales Human Research Ethics Committee 2021/ETH00988.

## Results

### Stages one and two: Identifying high-performing services and developing the implementation framework and integrating it into PCOC’s education program

A total of 15 services (8%) met the definition of a high performing service and six were selected for in-depth case studies. The selected services represented inpatient and community care settings. As a result of the case studies, 39 factors were identified that predispose, enable, and reinforce the integration of outcome measures as a tool for improving the quality of care. These factors were grouped into five overarching strategies resulting in version 1 of the framework. Following consultations with managers clinical leads and PCOC improvement facilitators, modifications were made to clarify, refine, and synthesize the factors to a list of 25, resulting in version 2 of the framework. Several factors were removed and included in a readiness assessment, enhancing support for services in the predisposing phase of implementation. The five key strategies include:

- Active leadership and clinical championing of PCOC.
- Continuous education of the clinical team to use the assessment and response models.
- Improved data literacy to enable patient outcome data to inform decision-making and quality improvement.
- Infrastructure to ensure the meaningful capture, transfer, and communication of outcome data; and
- Embedding PCOC into quality systems and processes within the palliative care service.

**FIG. 1.**
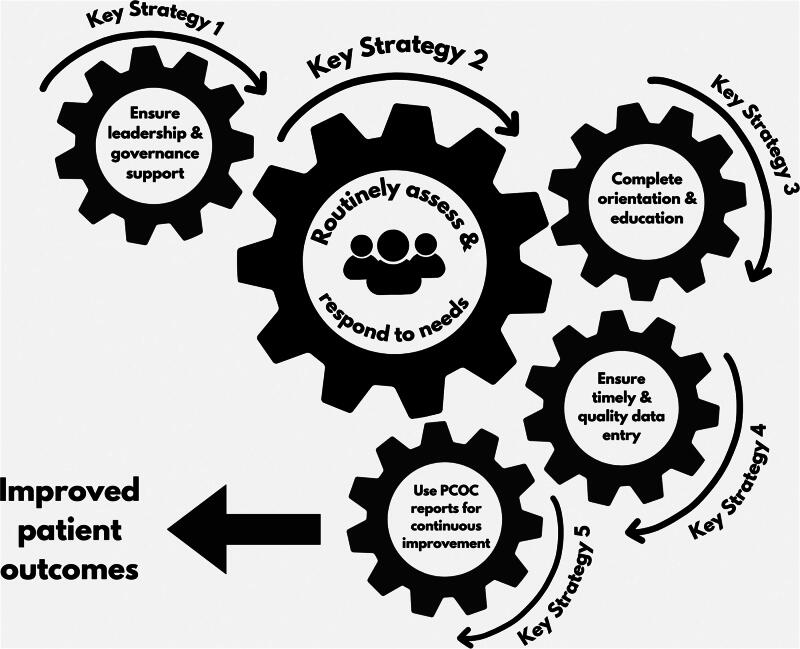
PCOC assessment and response model,^31^ with associated key strategies for implementation. PCOC, palliative care outcomes collaboration.

A summarized version of the framework to support the central assessment and response model for PCOC is shown in Table 1. The full version is available as Supplementary Data where additional information and mapping for the 14 factors are removed from the original list of 39. This framework titled “Key Strategies and Enabling Factors” includes a corresponding self-assessment tool that was developed to assist teams to continuously evaluate their implementation progress (available here). Central to the framework is high-quality assessment of patient and family needs and communication of these needs within the multidisciplinary team. This is represented by Figure 1 and described in a clinical assessment and response protocol.^31^

**Table 1.** Key Strategies for Implementation of the Assessment and Response Model (Summarized)

Strategy 1: Leadership and governance support for PCOC is secured, both at an organizational and service level (including the appropriate medical, nursing, and allied health leads, and quality managers). High-performing services have the support of leadership, clear governance, and accountability processes in place to ensure PCOC is meaningfully integrated into care delivery and quality improvement systems.
Strategy 2: Local processes are in place to support routine and multidisciplinary assessment and response. High-performing services have local policies and processes in place and network with their peers to ensure the meaningful application of the PCOC assessment and response model including participation of patients and family/carers in outcome measures.
Strategy 3: PCOC is included in orientation and ongoing training for medical, nursing, and allied health staff. High-performing services systematically and regularly engage in education to optimize routine outcome measurement for patients, health care professionals, and the organization. In addition, staff members are identified to lead a local education program for patients and staff on the use of PCOC, specifically the meaningful use of the assessment tools.
Strategy 4: Local infrastructure to support PCOC data entry, extraction, and quality are in place. High-performing services have data infrastructure to ensure data is of sufficient standard to be included in the national benchmarked results and can be used for quality improvement. The infrastructure ensures outcome data can be communicated within and between services.
Strategy 5: Continuous improvement through embedding quality improvement into systems and processes High-performing services incorporate PCOC data into quality systems and processes, including monitoring and evaluation. Identified staff members use the outcome results to improve clinical processes and clinical interventions. PCOC data is readily available and accessible to staff and the service engages in regular review of their outcome data.

PCOC, palliative care outcomes collaboration.

The “Key Strategies and Enabling Factors” framework was structured and integrated into the PCOC education program as summarized in Table 1. The education program can be described as a complex set of targeted interventions and resources. A summary of the education program incorporating the implementation framework is publicly available.^32^

### Phase three: preliminary analysis of the implementation framework

The post-intervention group included 11 services representing 8902 Patients and 24,563 Phases. There were eight inpatient palliative care services and five community services. The pre-intervention group had 20 services, nine more than the post-intervention group (Table 2). Just over half of the services in the pre-intervention group were medium sized (52.4%) whereas half of the services in the post-intervention group were large (Table 2).

**Table 2.** Summary of Service Characteristics in the Pre- and Post-Intervention Groups

Service characteristics	Pre-intervention *n* (%)	Post-intervention *n* (%)
Volume		
Services	20	11
Patient (across 2 years)	17,110	8902
Phases (across 2 years)	57,690	24,563
Care setting		
Inpatient services	14 (60.9)	8 (61.5)
Community services	9 (39.1)	5 (38.5)
Size of service (average admissions per year)		
Small (<100)	4 (19.0)	2 (16.7)
Medium (100–299)	11 (52.4)	4 (33.3)
Large (300+)	6 (28.6)	6 (50.0)
Patient characteristics		
Malignant diagnosis	74.4%	57.3%
Median AKPS at admission	40	40

AKPS, Australia-modified Karnofsky Performance Scale, the pre-intervention group includes PCOC services that received their first report between 2016 and 2019. The post-intervention group includes PCOC services that received their first report between 2022 and 2023. PCOC, palliative care outcomes collaboration.

All the benchmarks measuring the percent of phases starting moderate to severe and ending absent or mild were 10.8% to 26.9% higher after 6 months of PCOC participation (first benchmarking report) in the post-intervention group (pain and psychological/spiritual—PCPSS) (Fig. 1). The pre- and post-intervention groups showed similar trends after 6 months and over time in their benchmark outcomes in number of phases starting and ending absent or mild. An improvement plateau at the 18-month benchmarking period was evident in both groups. This phenomenon may be related to sustaining continuous improvement, which requires specific interventions.^33,34^

**FIG. 2.**
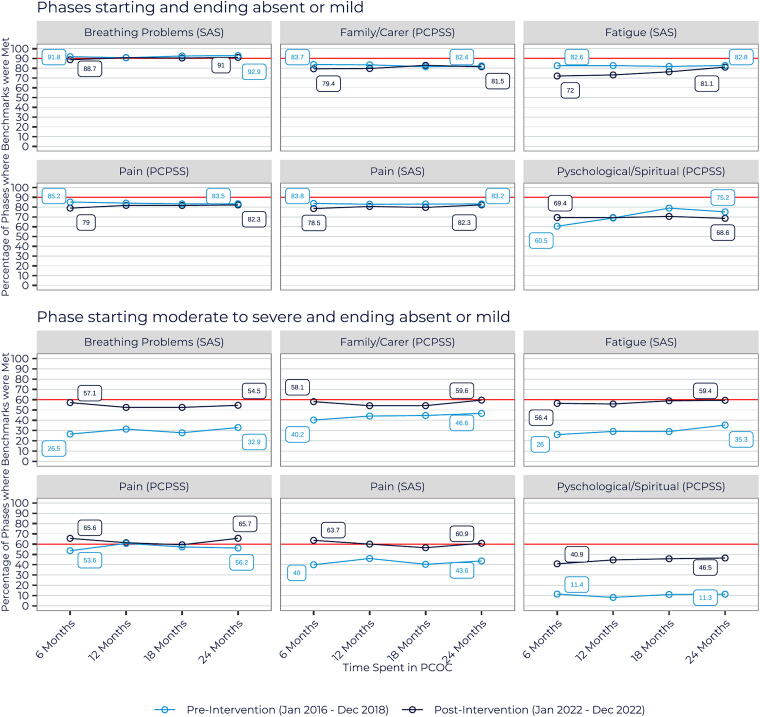
Percent of palliative care phases that met PCOC benchmarks in new PCOC services pre- and post-intervention. PCOC, palliative care outcomes collaboration.

There was little change in the difference in the percent of benchmarks met after 2 years in the pre and post-intervention group. The post-intervention group demonstrated an improvement in six of the 12 benchmarks. The largest difference was seen in the percentage of patients that reduced their fatigue (SAS) from moderate to severe to absent or mild during a phase (8.7%) (Table 3). The psychological and spiritual domain remained the farthest from achieving benchmarks in both the pre- and post-intervention groups (Fig. 2). In terms of pain management, little change was observed over 2 years in both groups across all measures, except in the pre-intervention group, where patients who entered with moderate to severe pain showed some improvement (Table 3).

**Table 3.** Difference in Percent of Palliative Care Phases that Met the PCOC Benchmarks in Report 1 and Report 4 After Implementation in the Pre- and Post-Intervention Groups

	Difference in percent of palliative care phases that met the PCOC benchmarks after 2 years (%)		Differences in pre- and post-intervention (%)
	Pre-intervention	Post-intervention	
Phases starting and ending absent or mild			
Breathing problems (SAS)	0.2	1.9	1.78
Family/carer (PCPSS)	2.1	−0.1	−2.16
Fatigue (SAS)	1.0	5.0	3.99
Pain (PCPSS)	1.1	2.0	0.92
Pain (SAS)	−0.8	2.9	3.79
Psychological/spiritual (PCPSS)	6.6	2.7	−3.90
Phase starting moderate to severe and ending absent or mild			
Breathing problems (SAS)	6.5	0.9	−11.12
Family/carer (PCPSS)	12.0	0.9	−2.47
Fatigue (SAS)	11.2	8.7	−6.64
Pain (PCPSS)	7.9	1.3	−7.55
Pain (SAS)	6.0	−1.5	−7.5
Psychological/spiritual (PCPSS)	−0.2	5.2	5.48

SAS, symptom assessment score; PCPSS, palliative care problem severity score; PCOC, palliative care outcomes collaboration.

## Discussion

Reviewing high-performing palliative care services enabled the development of an implementation framework consisting of five key strategies and 25 enabling factors. Our approach benefited from the maturity of the PCOC program with the framework aligning with findings from other studies of less established outcome initiatives.^35,36^ The framework has equipped the PCOC quality initiative with a structured yet adaptable approach to both implementation and education, based on services that have achieved sustained improvement in patient outcomes. By integrating the framework into the education program, we systematically prepared new services in embedding outcome measures into their organizational and interpersonal structures and processes and support services to be engaged in using their outcome data for quality improvement from their first report.

The preliminary results showed that services who received the new implementation framework as part of their PCOC onboarding were more capable of managing moderate to severe symptom distress in their patients within the first 6 months than those that did not receive the framework. While services post-implementation demonstrated improvements in symptom domains, challenges remain. Such challenges include the best approach to psycho-spiritual support. The complexity of this domain is likely influenced by variables that are not fully understood, such as a lack of available psycho-spiritual services in Australia,^37^ and the need for more specific assessment measures and education.^38^ Further research and investment in this area are critical to improving care outcomes in this often-overlooked aspect of palliative care. Similarly, there were minimal improvements in pain outcomes over time. However, some gains were observed in both pre- and post-intervention groups, despite high initial performance levels in the post-intervention group. Previous analyses over a 10-year period have reported that one in five cases of severe pain remained unresolved,^9^ highlighting the persistent challenge of achieving substantial improvements in pain for palliative care patients.

The new implementation framework and education program were provided to all participating services, yet we did not analyze the outcome results for improvement across all services or investigate the reason for variation between them. It is possible that some services require re-implementation due to failed or flawed implementation,^39^ and we did not explore this in our study. PCOC has previously been shown to be feasible on a national scale, with improvements in outcomes well-before the existence of our implementation framework.^7^ This may be attributed to critical success factors such as the role of the PCOC Improvement Facilitator, who works directly with services to activate implementation and knowledge translation.^6,40^

This study had several limitations. First, we were unable to examine all of the factors that influence patient outcomes. Although services that received the new implementation framework and education had a higher initial performance level and improved with participation, there are many factors that also influence patient outcomes. These factors include individual patient characteristics such as diagnosis, gender, and other demographic variables, as well as access-related factors such as the timing of referral and proximity to services.^41–43^ Service-level factors such as workforce availability, resources, and funding,^44–46^ also play a significant role in determining the quality of care and patient outcomes. Second, the preliminary analysis only included 11 services that had completed the onboarding process using the new implementation framework and received four reports to be analyzed. Future research should conduct a more comprehensive analysis of the framework once more data has been collected. Further analysis should also explore the links between successful implementation and outcome improvements.

## Conclusions

The integration of outcome measures has shown positive impacts on patient outcomes, but achieving comprehensive improvements in palliative care requires ongoing support, leadership, and targeted efforts in underperforming areas. Implementing routine outcome measurement at scale demands a structured approach, central to which is a standardized assessment and response model. The PCOC key strategies and enabling factors framework emphasizes the balance across systems and structures, with adaption to local contexts to support quality improvement in palliative care through the seamless integration of routinely collected outcome measures into all facets of clinical care and service delivery.

## Abbreviations Used

AKPS: Australia-modified Karnofsky Performance Scale
COVID-19: Coronavirus
PCOC: Palliative Care Outcomes Collaboration
PCPSS: Palliative Care Problem Severity Score
RUG-ADL: Resource Utilisation Groups—Activities of Daily Living
SAS: Symptom Assessment Scale
SQUIRE 2.0: Standards for Quality Improvement Reporting Excellence
