# Supplementary material for: A National Quality Initiative to Improve Palliative Care Outcomes: Identifying Enabling Factors that Drive Quality Improvement
Source: Palliat Med Rep. 2025 May 9;6(1):241–50. doi: 10.1089/pmr.2024.0092 (PMC12410334; doi:10.1089/pmr.2024.0092)
Supplement: Supplementary Data [file pmr.2024.0092_supplementary_data.docx]

**Supplementary file 1: Key strategies and enabling factors version 1 and version 2 mapped.**

Version 2 includes 25 factors. Version 1 included 35 factors. These factors were either merged with existing factors or moved over to a readiness assessment. The readiness assessment is used pre implementation to determine if the palliative care service is ready for quality improvement to start using outcome measures and benchmarking. The readiness assessment can be found on the PCOC [website](https://documents.uow.edu.au/content/groups/public/@web/@chsd/@pcoc/documents/doc/uow275157.pdf)

| **Version 2: key strategies and enabling factors** |
| --- |
| **Key Strategy 1: Leadership support for PCOC is secured, both at an organisational and service level** |
| 1. Provide direction on how to use PCOC reports |
| 2. Attend benchmarking workshops and/or PCOC events |
| 3. Provide opportunities for staff to participate in PCOC education |
| 4. Ensure the PCOC Assessment and Response protocol is included in relevant policies and procedures |
| **Key Strategy 2: Routine assessment and response** |
| 1. All five assessment tools are used to routinely assess patients |
| 2. Assessments are documented at point of care |
| 3. Clinicians have access to the full PCOC assessment definitions |
| 4. Patients and family are given the SAS tool to help rate symptom distress |
| 5. PCOC scores form part of clinical handover |
| 6. Assessment scores are used to help guide and plan patient care |
| 7. PCOC scores are discussed in care planning and patient review meetings |
| 8. All staff (including medical, nursing, allied health, pastoral care) use PCOC tools as part of patient assessment |
| **Key Strategy 3: PCOC data entry, extraction and quality** |
| 1. Data errors are identified and corrected |
| 2. Adequate time is allocated to ensure accurate data entry |
| **Key Strategy 4: Orientation and education** |
| 1. Orientation includes PCOC assessment and response protocol |
| 2. PCOC is regularly included in in-service education |
| 3. PCOC lanyard cards are provided to staff |
| 4. PCOC Essentials online course is completed by staff |
| 5. PCOC champions and clinical leads attend relevant PCOC workshops |
| 6. All staff (including medical, nursing, allied health, pastoral care) participate in ongoing PCOC education |
| **Key Strategy 5: Quality improvement and service development** |
| 1. PCOC reports are used to identify areas for quality improvement, service development and/or research |
| 2. Audit tools are used to ensure the accuracy and reliability of assessments |
| 3. PCOC case reviews are used as part of an improvement strategy |
| 4. Staff attend benchmarking and/or advanced workshops |
| 5. PCOC is integrated into quality systems (e.g. accreditation processes) |

| **Version 1: factors mapped across to version 2 or into readiness assessment** | |
| --- | --- |
| **Key Strategy 1: Leadership support for PCOC is secured, both at an organisational and service level** | |
| Key leadership staff provide direction and support to routinely and systematically assess patients | Readiness Assessment |
| Leadership staff provide feedback to staff about PCOC performance | Version 2 Key Strategy 5, Factor 1 |
| Service has identified a ‘Service Facilitator’ (PCOC champion) to be a key contact and resource person | Readiness Assessment |
| Staff are provided with opportunities to attend Fundamentals Workshops or refresher training as part of their work | Version 2 Key Strategy 4, Factors 2,3,4,5,6 |
| Accreditation is aligned with PCOC (see PCOC/national standards tool). | Version 2 Key Strategy 5, Factor 5 |
| Feedback on patient outcome results is included as a standing item on management meeting agendas. | Readiness Assessment |
| A system of planning quality improvement based on patient outcomes results is established. | Version 2 Key Strategy 5, Factor 5 |
| Patient outcomes reports inform service planning for delivery of care. | Version 2 Key Strategy 5, Factor 1 |
| **Key Strategy 2: Routine assessment and response** | |
| Patient referrals are triggered by assessment scores | Version 2 Key Strategy 2, Factor 6 |
| Allied health staff utilise the PCOC tools to communicate with staff and assess and respond to patient needs. | Version 2 Key Strategy 2, Factor 8 |
| The PCOC Assessment Protocol is used to inform the development of the assessment protocol / framework at the team level. | Version 2 Key Strategy 2, Factor 6 |
| The assessment protocol at the team level for response to symptoms and problems incorporates assessment scores. | Version 2 Key Strategy 2, Factor 6 |
| **Key Strategy 3: PCOC data entry, extraction and quality** | |
| Nil |  |
| **Key Strategy 4: Orientation and education** | |
| Allied health staff participate in PCOC orientation and in-service programs and attend PCOC Fundamentals Workshops | Version 2 Key Strategy 4, Factor 6 |
| **Key Strategy 5: Quality improvement and service development** | |
| Outcome results are shared with others for the purpose of mentoring, capacity building and collaboration. | Version 2 Key Strategy 5, Factor 1 |
